# The impact of exogenous vitamin D on thyroid autoimmunity in euthyroid men with autoimmune thyroiditis and early-onset androgenic alopecia

**DOI:** 10.1007/s43440-021-00295-3

**Published:** 2021-06-09

**Authors:** Robert Krysiak, Karolina Kowalcze, Bogusław Okopień

**Affiliations:** 1 Department of Internal Medicine and Clinical Pharmacology, Medical University of Silesia, Medyków 18, 40-752 Katowice, Poland; 2 Department of Pediatrics in Bytom, School of Health Sciences in Katowice, Medical University of Silesia, Katowice, Poland

**Keywords:** Androgenic alopecia, Hypothalamic-pituitary-thyroid axis, Thyroid autoimmunity, Vitamin D

## Abstract

**Background:**

Early-onset androgenic alopecia is regarded as the phenotypic equivalent of polycystic ovary syndrome in men. Women with polycystic ovary syndrome are at high risk of autoimmune thyroiditis. The aim of the current study was to investigate whether early-onset androgenic alopecia determines the impact of exogenous vitamin D on thyroid autoimmunity and thyroid function in men with autoimmune thyroiditis.

**Methods:**

The study included 67 young men with autoimmune thyroiditis, 25 of whom had early-onset androgenic alopecia (group A). All 25 men with alopecia and 23 out of the 42 men with no evidence of hair loss, matched for age, antibody titers and thyrotropin levels (group B), were then treated with vitamin D (100 μg daily). Serum titers of thyroid peroxidase and thyroglobulin antibodies, serum levels of thyrotropin, free thyroid hormones, total and calculated free testosterone, dehydroepiandrosterone-sulfate, estradiol, prolactin and 25-hydroxyvitamin D, as well as the calculated parameters of thyroid homeostasis were assessed before vitamin D treatment and 6 months later.

**Results:**

At baseline, thyroid antibody titers were higher in subjects with than without alopecia and correlated with calculated free testosterone levels. Vitamin D reduced antibody titers in both groups but this effect was stronger in group B than group A. Only in group B, vitamin D increased SPINA-GT. The impact of vitamin D on antibody titers correlated with 25-hydroxyvitamin D levels, calculated free testosterone, treatment-induced increase in 25-hydroxyvitamin D levels and the improvement in insulin sensitivity.

**Conclusion:**

This study suggests that euthyroid men with early-onset androgenic alopecia may benefit to a lesser degree from vitamin D treatment than other subjects with autoimmune thyroiditis.

## Introduction

Autoimmune (Hashimoto’s) thyroiditis is the most prevalent organ-specific autoimmune disorder and the leading cause of hypothyroidism in developed countries [1]. Cell- and antibody-mediated immune processes in this disorder result in the replacement of thyroid parenchyma by lymphocytes, and at later stages, also by fibrous tissue [2]. Despite a higher prevalence in women, more and more men are being diagnosed with this disorder [1, 2].

Androgenic alopecia is considered the most common form of hair loss in men, affecting over 30% of individuals by the age of 30 and 80% of men during their lifespan [3, 4]. The disease preferentially affects the temples, vertex and mid-frontal scalp, leaving a rim of hair at the sides and sparing the occipital region [3]. Early-onset androgenic alopecia is regarded as the male equivalent of polycystic ovary syndrome (PCOS) [5, 6]. Compared with age-matched men with no evidence of hair loss, individuals with early-onset male pattern hair loss were characterized by increased levels of androgens (testosterone and dehydroepiandrosterone-sulfate [DHEA-S]), prolactin and luteinizing hormone (LH), decreased levels of follicle-stimulating hormone (FSH) and sex hormone-binding globulin, as well as increased values of the LH/FSH ratio and the free androgen index [7]. There is a strong association between early-onset androgenic alopecia in men, particularly in Europeans, and a risk of development of metabolic syndrome [8]. Finally, early-onset male pattern hair loss may predispose to obesity, insulin resistance, dyslipidemia, hypertension and atherosclerosis [6, 9]. Interestingly, both men with androgenic alopecia and male siblings of women with PCOS were found to have lower levels of 25-hydroxyvitamin D than control subjects [10, 11].

It seems that women with PCOS are characterized by a higher prevalence of autoimmune (Hashimoto’s) thyroiditis and elevated titers of thyroid antibodies than women without PCOS [12]. The link between both disorders may be partially explained by the impact of hormonal and metabolic factors [12]. However, most women with PCOS are treated because of hyperandrogenism, anovulation, amenorrhea/oligomenorrhea and/or insulin resistance, which makes it difficult to study the causal relationship between both disorders. Recently, it has been shown that exogenous dehydroepiandrosterone exerts a beneficial effect on thyroid autoimmunity and hypothalamic-pituitary-thyroid axis activity in men with autoimmune hypothyroidism and low DHEA-S levels [13], exogenous testosterone has a protective effect on thyroid autoimmunity in men with Hashimoto's thyroiditis and testosterone deficiency [14], and that testosterone therapy enhances the beneficial effect of vitamin D on thyroid autoimmunity and thyroid secretory function in men with testosterone deficiency [15]. These findings suggest that androgen levels may affect thyroid autoimmunity and functioning in men. However, to the best of our knowledge no previous study investigated the association between early-onset androgenic alopecia and treatment of Hashimoto’s thyroiditis in men. Therefore, the aim of the present study was to investigate whether the presence of early-onset androgenic alopecia determines the impact of vitamin D on thyroid autoimmunity in men.

## Materials and methods

The study protocol was approved by the institutional review board (the Bioethical Committee of the Medical University of Silesia [KNW/0022/KB/162/16]; July 5, 2016) and all participants gave written, informed consent after receiving a complete description of the study. All procedures were performed according to the research ethics of the Declaration of Helsinki. Because of the non-randomized nature of the study, as well as because the assignment of the medical intervention was not at the discretion of the investigators (all subjects received the same treatment), the study did not meet the criteria of a clinical trial and did not require prospectively registration prior to patient enrollment.

### Patients

The participants of the current study were selected among young men (aged 18–35 years old) who had been screened at our Department for the presence of thyroid disease. To be included, they were required to meet the following criteria: (a) serum titers of thyroid peroxidase antibodies (TPOAb) above 100 U/ml; (b) a diffusely enlarged hypoechoic gland or multiple hypoechoic foci in the thyroid parenchyma on thyroid sonography; (c) serum thyrotropin levels in the range between 0.4 and 4.5 mU/L; as well as (d) serum-free thyroid hormone levels within the references range (free thyroxine levels between 10.0 and 21.0 pmol/L and free triiodothyronine between 2.2 and 6.5 pmol/L).

A sample size analysis showed that assuming a power of 80% and a significance level of 0.05, at least 21 individuals had to be included into each group to detect a 20% difference between both groups in titers of thyroid antibodies (the primary endpoint). The study consisted of two parts. The first one included 25 men with early-onset androgenic alopecia (group A) and 42 men with no evidence of hair loss, serving as a control group. Early-onset androgenic alopecia was defined as grade 3 vertex or more alopecia on the Hamilton and Norwood scale (used to classify the stages of male pattern baldness [16, 17]) by the age of 30. To minimize the impact of seasonal fluctuations in antibody titers, 25-hydroxyvitamin D and hormone levels, 35 men were included in January and February, while the remaining 32 patients were enrolled in July or August. All subjects from group A, as well as 23 out of the 42 individuals with no evidence of hair loss (group B), selected from the control group, participated also in the second part of the study. The selection procedure, performed using the freely available PEPI-for-Windows computer program, was intended to obtain two groups matched for age, thyroid antibody titers and thyrotropin levels. Thirteen men from group A and 12 men from group B were recruited in winter, while 12 men from group A and 11 men from group B were recruited in summer.

The subjects were excluded if they met at least one of the following criteria: positive serum antibodies against thyrotropin receptor, other autoimmune or endocrine disorders, impaired renal or liver function, malabsorption syndrome, body mass index above 35 kg/m^2^, any other serious disorder, any pharmacotherapy and poor compliance.

### Study design

Over the entire study period (six months), the participants were treated with exogenous vitamin D (100 μg [4000 IU] daily), administered in the morning (between 7.30 and 8.30 a.m.). Participants were seen every 6 weeks to ensure adherence to vitamin D treatment and to boost compliance with the study protocol. Compliance was assessed by tablet counts and analysis of individual dietary questionnaires. Medication adherence was assessed by means of a four-item Morisky-Green test and pill count, while daily dietary vitamin D intake by analysis of individual dietary questionnaires.

### Laboratory assays

All laboratory assays were performed in duplicate (to ensure the consistency of assessments) at the beginning of the study and 6 months later. Blood samples for laboratory were collected between 7.30 and 8.30 a.m. after 12-h overnight fasting in a quiet and air-conditioned room (constant temperature of 23–24 °C). Prior to venipuncture, all patients had been resting for at least 30 min in the seated position. Serum titers of TPOAb and thyroglobulin antibodies (TgAb), as well as serum levels of thyrotropin, free thyroxine, free triiodothyronine, DHEA-S, total testosterone, estradiol, sex-hormone binding globulin, prolactin, insulin and 25-hydroxyvitamin D were assayed by direct chemiluminescence using acridinium ester technology (ADVIA Centaur XP Immunoassay System, Siemens Healthcare Diagnostics, Munich, Germany). Plasma glucose and albumin concentrations were measured by routine laboratory techniques using commercially available kits (Roche Diagnostics, Basel, Switzerland). The structure parameters of thyroid homeostasis (Jostel’s thyrotropin index, SPINA-GT and SPINA-GD) were calculated on the basis of thyrotropin and free thyroid hormone levels as previously described [18, 19] using the online freely available SPINA-Thyr 4.0.1 for Mac Universal software. Free testosterone was calculated based on testosterone and sex hormone-binding globulin levels using the online calculator, which is available at www.issam.ch/freetesto.htm. The homeostasis model assessment 1 of insulin resistance index (HOMA1-IR) was calculated as follows: fasting glucose (mg/dL) × fasting insulin (mIU/L)/405.

### Statistical analysis

Because of skewed distributions, all parameters were natural-log transformed to achieve normality and homogeneity of variance, as well as and were transformed back for reporting in the tables. Comparisons between the groups, as well as between changes from baseline after adjustment for baseline values (reflecting the strength of vitamin D action) were performed using Student’s *t*-test for independent samples. The differences between the means of variables within the same treatment group were analyzed with Student’s paired *t*-test. Categorical variables were analyzed by *χ*^2^ test. Correlations between the outcome variables were calculated using Pearson's *r*-tests. To find variables that have a significant effect on the changes in thyroid antibody titers, stepwise multivariate regression analysis with correction for multiple comparisons was carried out with treatment-induced changes in antibody titers as dependent variables and variables that were significantly correlated with the primary endpoint as independent variables. The results were regarded as statistically significant if two-tailed *p*-values corrected for multiple testing (using the Benjamini–Hochberg procedure) were below 0.05. All *p*-values shown in the Results section and in the tables are values adjusted for multiple comparisons.

## Results

### Part 1

There were no differences between the study groups in age, smoking, body mass index, systolic and diastolic blood pressure, serum levels of thyrotropin, free thyroid hormones and prolactin, as well as in Jostel’s thyrotropin index and SPINA-GD. Compared with the control group, men with early-onset androgenic alopecia showed higher titers of TPOAb and TgAb, higher concentrations of DHEA-S, testosterone and calculated free testosterone, lower levels of 25-hydroxyvitamin D, and higher mean values of HOMA1-IR and SPINA-GT (Table 1).

**Table 1:** Comparison between subjects with autoimmune thyroiditis and early-onset androgenic alopecia or normal hair growth

Variable	Subjects with early-onset androgenic alopecia	Subjects with normal hair growth	*p*-value [between both groups]
Number [*n*]	25	42	–
Age [years; mean (SD)]	27 (5)	28 (4)	0.3711
Smokers [%]	28	31	–
Body mass index [kg/m^2^; mean (SD)]	28.2 (4.1)	27.8 (3.5)	0.5977
Systolic blood pressure [mmHg; mean (SD)]	123 (10)	120 (8)	0.181480
Systolic blood pressure [mmHg; mean (SD)]	80 (5)	79 (5)	0.4314
TPOAb [IU/mL; mean (SD)]	955 (385)	762 (315)	**0.0292**
TgAb [IU/mL; mean (SD)]	894 (372)	724 (298)	**0.0438**
Thyrotropin [mIU/L; mean (SD)]	2.7 (0.8)	2.5 (0.6)	0.2490
Free thyroxine [pmol/L; mean (SD)]	14.9 (3.4)	16.1 (3.1)	0.1442
Free triiodothyronine [pmol/L; mean (SD)]	4.0 (0.7)	4.2 (0.7)	0.2622
Jostel’s thyrotropin index [mean (SD)]	3.0 (0.2)	3.1 (0.2)	0.074
SPINA-GT index [pmol/s; mean (SD)]	2.28 (0.35)	2.57 (0.37)	**0.0024**
SPINA-GD index [nmol/s; mean (SD)]	24.82 (2.65)	24.12 (2.14)	0.2409
Prolactin [ng/mL; mean (SD)]	11.0 (5.0)	10.2 (4.1)	0.4796
DHEA-S [µmol/L; mean (SD)]	4.7 (0.8)	4.0 (0.5)	** < 0.0001**
Total testosterone [nmol/L; mean (SD)]	20.8 (6.5)	17.5 (4.9)	**0.0171**
Calculated free testosterone [pmol/L; mean (SD)]	345 (68)	284 (58)	**0.0002**
Estradiol [pmol/L; mean (SD)]	150 (28)	142 (23)	0.2091
25-hydroxyvitamin D [ng/mL; mean (SD)]	29.2 (11.0)	36.8 (10.5)	**0.0064**
HOMA1-IR [mean (SD)]	3.2 (0.8)	2.8 (0.7)	**0.0358**

Comparisons between the groups were performed using Student’s *t*-test for independent samples. Categorical variables were analyzed by *χ*^2^ test. Statistically significant results are marked in bold

Baseline antibody titers correlated with thyrotropin levels (TPOAb: *r* = 0.46 [*p* = 0.0004], TgAb: *r* = 0.41 [*p* = 0.0008]), calculated free testosterone (TPOAb: *r* = 0.46 [*p* = 0.0001], TgAb: *r* = 0.41 [*p* = 0.0006]), 25-hydroxyvitamin D levels (TPOAb: *r* = − 0.37 [*p* = 0.0047], TgAb: *r* = − 0.30 [*p* = 0.0325]) and SPINA-GT (TPOAb: *r* = − 0.29 [*p* = 0.0218], TgAb: *r* = − 0.25 [*p* = 0.0421]). Moreover, TPOAb titers correlated with total testosterone (TPOAb: *r* = 0.34 [*p* = 0.0084) and DHEA-S (TPOAb: *r* = 0.30 [*p* = 0.0387]).

### Part 2

Before the treatment period, groups A and B were comparable with respect to age, body mass index, smoking, HOMA1-IR, serum titers of thyroid antibodies, serum levels of thyrotropin, free thyroid hormones, DHEA-S, total testosterone, prolactin and estradiol, and all calculated parameters of thyroid homeostasis. Calculated free testosterone levels were higher in group A than group B.

No serious adverse effects were reported during the study period and all patients completed the study. The analysis of eating diaries showed that the average daily dietary vitamin D intake (not counting the amount of vitamin D contained in vitamin D preparations) was similar in both study groups (group A: 426 (185) IU; group B: 419 (164)).

Vitamin D reduced TPOAb and TgAb titers, as well as increased 25-hydroxyvitamin D in both groups but these effects were stronger in group B than group A. Only in group B, vitamin D increased SPINA-GT and reduced calculated free testosterone and HOMA1-IR. In both groups, serum levels of thyrotropin, free thyroxine, free triiodothyronine, DHEA-S, total testosterone, estradiol and prolactin, as well as Jostel’s thyrotropin index and SPINA-GD remained at a similar level throughout the study. At the end of the study period, both groups differed from each other in titers of thyroid antibodies, levels of 25-hydroxyvitamin D and calculated free testosterone, HOMA1-IR and SPINA-GT (Table 2).

**Table 2:** The effect of vitamin D on thyroid antibody titers, hormones, insulin sensitivity and calculated parameters of thyroid homeostasis in men with or without early-onset androgenic alopecia and autoimmune thyroiditis

Variable	Group A^a^ (*n* = 25)	Group B^b^ (*n* = 23)	*p*-value [Group A vs. Group B]
TPOAb [IU/mL; mean (SD)]			
At the beginning of the study	955 (385)	908 (361)	0.6654 **0.0430**
At the end of the study	709 (268)	564 (208)*	
*p*-value [post-treatment vs. baseline]	**0.0117**	**0.0003**	–
TgAb [IU/mL; mean (SD)]			
At the beginning of the study	894 (372)	868 (320)	0.7668
At the end of the study	701 (280)	551 (208)*	**0.0420**
*p*-value [post-treatment vs. baseline]	**0.0396**	**0.0003**	–
Thyrotropin [mIU/L; mean (SD)]			
At the beginning of the study	2.7 (0.8)	2.6 (1.0)	0.7027
At the end of the study	2.7 (1.0)	2.3 (0.8)	0.1350
*p*-value [post-treatment vs. baseline]	1.0000	0.2373	–
Free thyroxine [pmol/L; mean (SD)]			
At the beginning of the study	14.9 (3.4)	15.2 (2.8)	0.7414
At the end of the study	14.7 (4.2)	17.0 (3.7)	0.0508
*p*-value [post-treatment vs. baseline]	0.8239	0.0695	–
Free triiodothyronine [pmol/L; mean (SD)]			
At the beginning of the study	4.0 (0.7)	4.1 (0.8)	0.6464
At the end of the study	4.2 (0.8)	4.5 (1.0)	
*p*-value [post-treatment vs. baseline]	0.3516	0.1413	
Jostel’s thyrotropin index [mean (SD)]			
At the beginning of the study	3.0 (0.2)	3.0 (0.2)	1.0000
At the end of the study	3.0 (0.2)	3.1 (0.2)	0.2552
*p*-value [post-treatment vs. baseline]	1.0000	0.0970	–
SPINA-GT index [pmol/s; mean (SD)]			
At the beginning of the study	2.28 (0.35)	2.37 (0.40)	0.4101
At the end of the study	2.25 (0.41)	2.83 (0.42)*	** < 0.0001**
*p*-value [post-treatment vs. baseline]	0.7820	**0.0004**	–
SPINA-GD index [nmol/s; mean (SD)]			
At the beginning of the study	24.82 (2.65)	24.94 (3.48)	0.8931
At the end of the study	26.42 (3.85)	24.48 (3.79)	0.0856
*p*-value [post-treatment vs. baseline]	0.09344	0.6702	–
Prolactin [ng/mL; mean (SD)			
At the beginning of the study	11.0 (5.0)	10.8 (4.9)	0.8894
At the end of the study	10.0 (4.3)	9.7 (4.6)	0.8164
*p*-value [post-treatment vs. baseline]	0.4540	0.4367	–
DHEA-S [µmol/L; mean (SD)]			
At the beginning of the study	4.7 (0.8)	4.3 (0.8)	0.0902
At the end of the study	4.4 (0.9)	4.3 (1.0)	0.7170
*p*-value [post-treatment vs. baseline]	0.2189	1.0000	–
Total testosterone [nmol/L; mean (SD)]			
At the beginning of the study	20.8 (6.5)	18.6 (5.0)	0.1981
At the end of the study	19.5 (5.9)	16.3 (5.5)	0.0587
*p*-value [post-treatment vs. baseline]	0.4626	0.1449	–
Calculated free testosterone [pmol/L; mean (SD)]			
At the beginning of the study	345 (68)	302 (56)	**0.0215**
At the end of the study	310 (61)	251 (60)*	**0.0015**
*p*-value [post-treatment vs. baseline]	0.0614	**0.0047**	–
Estradiol [pmol/L; mean (SD)]			
At the beginning of the study	150 (28)	139 (31)	0.2029
At the end of the study	144 (35)	137 (26)	0.4388
*p*-value [post-treatment vs. baseline]	0.5065	0.8137	–
25-hydroxyvitamin D [ng/mL; mean (SD)]			
At the beginning of the study	29.2 (11.0)	34.1 (10.6)	0.1236
At the end of the study	35.0 (7.1)	46.1 (12.5)*	**0.0004**
*p*-value [post-treatment vs. baseline]	**0.0315**	**0.0010**	–
HOMA1-IR [mean (SD)]			
At the beginning of the study	3.2 (0.8)	3.0 (0.8)	0.3914
At the end of the study	3.1 (0.8)	2.5 (0.7)*	**0.0077**
*p*-value [post-treatment vs. baseline]	0.6605	**0.0291**	–

Comparisons between the groups, as well as between changes from baseline after adjustment for baseline values (reflecting the strength of vitamin D action) were performed using Student’s *t*-test for independent samples. The differences between the means of variables within the same treatment group were analyzed with Student’s paired *t*-test. Statistically significant results are marked in bold

*The impact of exogenous vitamin D [percent changes from baseline after adjustment for baseline values] stronger than in the second group

^a^Men with early-onset androgenic alopecia

^b^Control men

The impact of vitamin D on antibody titers correlated with their baseline values (Group A—TPOAb: *r* = 0.62 [*p* < 0.0001], TgAb: *r* = 0.55 [*p* < 0.0001]; Group B—TPOAb: *r* = 0.58 [*p* < 0.0001], TgAb: *r* = 0.53 [*p* = 0.0317]), baseline 25-hydroxyvitamin D levels (Group A—TPOAb: *r* = 0.32 [*p* = 0.0118], TgAb: *r* = 0.25 [*p* = 0.0456]; Group B—TPOAb: *r* = 0.35 [*p* = 0.0104], TgAb: *r* = 0.28 [*p* = 0.0317]), calculated free testosterone (Group A—TPOAb: *r* = − 0.38 [*p* = 0.0035], TgAb: *r* = − 0.34 [*p* = 0.0088]; Group B—TPOAb: *r* = − 0.40 [*p* = 0.0008], TgAb: *r* = − 0.31 [*p* = 0.0204]), treatment-induced increase in 25-hydroxyvitamin D levels (Group A—TPOAb: *r* = 0.40 [*p* = 0.0010], TgAb: *r* = 0.35 [*p* = 0.0075]; Group B—TPOAb: *r* = 0.42 [*p* = 0.0007], TgAb: *r* = 0.38 [*p* = 0.0024]) and in group B with the improvement in insulin sensitivity (TPOAb: *r* = 0.29 [*p* = 0.0386], TgAb: *r* = 0.25 [*p* = 0.0461]). Treatment-induced changes in TPOAb correlated also with baseline values of DHEA-S (Group A—*r* = -− 0.24 [*p* = -0.0474]; Group B—*r* = − 0.28 [*p* =  − 0.0305]) and total testosterone (Group A—*r* = − 0.29 [*p* = − 0.0205]; Group B—*r* = − 0.31 [*p* = − 0.0183]). Moreover, treatment-induced changes in TPOAb in group B correlated with the effect of treatment on calculated free testosterone (*r* = 0.38 [*p* = 0.0040]) and on SPINA-GT (*r* = 0.30 [*p* = 0.0281]). Stepwise multiple regression analysis showed that baseline antibody titers, baseline 25-hydroxyvitamin D levels, baseline calculated free testosterone, treatment-induced changes in 25-hydroxyvitamin D levels and treatment-induced changes in HOMA1-IR were independently and significantly associated with the impact of vitamin D on thyroid antibody titers (Table 3).

**Table 3:** Stepwise multivariate regression analysis for treatment-induced changes in thyroid antibody titers

	ΔTPOAb			ΔTgAb		
	*β*	Partial *R*^2^	*p*-value	*β*	Partial *R*^2^	*p*-value
Baseline titers	0.425	0.202	< 0.0001	0.403	0.196	< 0.0001
Baseline 25-hydroxyvitamin D levels	0.295	0.104	0.0064	0.241	0.088	0.0096
Baseline calculated free testosterone	− 0.308	0.124	0.0008	− 0.295	0.092	0.0084
Δ25-hydroxyvitamin D levels	0.358	0.154	0.0002	0.314	0.125	0.0007
ΔHOMA1-IR	0.255	0.008	0.0124	0.228	0.0061	0.0223

Model *R*^2^ for ΔTPOAb = 0.644; Model *R*^2^ for ΔTgAb = 0.562

## Discussion

In line with previous studies [7, 8], men with early-onset androgenic alopecia were characterized by increased androgen levels and impaired insulin sensitivity. Unexpectedly, although the study included only subjects with elevated titers of TPOAb and TgAb, these titers were higher in individuals with early-onset androgenic alopecia than in control subjects. Titers of thyroid antibodies also correlated with androgen levels. Free testosterone levels calculated by Vermeulen's equation, used in the current study, correlates to a high degree with concentrations of free testosterone measured using equilibrium dialysis. This parameter had the strongest correlations with antibody titers, which probably resulted from the fact that only unbound hormone binds the androgen receptor in target tissues and exerts its action [20, 21]. Therefore, differences in thyroid antibody titers between men with and without alopecia are probably associated with a direct action of testosterone on the androgen receptor and do not seem to be mediated by aromatization to estradiol. Higher titers of thyroid antibodies in subjects with higher androgen concentrations are in contrast with previous observations that testosterone and dehydroepiandrosterone replacement therapy in men with subnormal levels of these hormones and coexisting Hashimoto’s thyroiditis reduced thyroid autoimmunity and slightly improved thyroid function [13, 14]. There are different explanations for this discrepancy. Participants of the present study were younger than subjects included in previous ones. Apart from thyroiditis and alopecia, they did not have any coexisting disorders or receive any chronic treatment. Another explanation are differences in the baseline hormone status. Unlike men with late-onset hypogonadism [14] and men with impaired functioning of the adrenal reticular zone [13], the current study included subjects with normal or high-normal androgen levels. Interestingly, both low and high androgen status may predispose to autoimmune thyroid diseases. Men with Klinefelter syndrome, the most frequent form of male hypogonadism, are often diagnosed with Hashimoto’s thyroiditis [22], while the (CAG)n repeat polymorphism, determining the sensitivity of the androgen receptor, correlated with the age of onset of Hashimoto’s thyroiditis [23]. Moreover, high amounts of androgens were found to stimulate Th cells to produce type 1 cytokines, playing a role in autoimmune thyroid destruction and contributing to the development and progression of autoimmune thyroiditis [24]. Our findings cannot be attributed to the conversion of androgens to estrogens because there were no between-group differences in estradiol levels, as well as because estradiol levels did not correlate with thyroid antibodies. Finally, the obtained results may be a consequence of low vitamin D status, which is frequently observed in subjects with autoimmune thyroid disorders [25]. In line with this finding, 25-hydroxyvitamin D levels were lower in individuals with alopecia, and correlated with titers of TPOAb and TgAb. Low 25-hydroxyvitamin D levels may be a consequence of impaired cholecalciferol production or of a stimulatory effect of androgens on 1α-hydroxylase activity, which is a key enzyme in cholecalciferol metabolism that converts 25-hydroxyvitamin D to calcitriol [26].

The second important finding of our study is that men with early-onset androgenic alopecia and coexistent autoimmune thyroiditis differed from individuals with normal hair growth in a less pronounced decrease in thyroid antibody titers. Moreover, only in the control subjects, vitamin D increased SPINA-GT, one of three major calculated parameters of thyroid homeostasis. These parameters, describing constant properties of the overall feedback control system, are calculated from circulating hormone levels obtained in vivo based on equilibrium analysis of a compartmental nonlinear model [19]. SPINA-GT estimates secretory capacity of the thyroid gland with higher sensitivity than thyrotropin and total or free thyroid hormones and may be regarded as a marker of mild thyroid hypofunction, even in subjects with thyrotropin and thyroid hormone levels within the reference range [19]. Therefore, the obtained findings allow us to assume that exogenous cholecalciferol-induced reduction in thyroid autoimmunity may slightly improve the function of the thyroid gland in patients with normal hair growth but does not seem to affect the secretory capacity of this gland in men with early-onset alopecia. These findings seem to be clinically relevant because early-onset androgenic alopecia can be easily diagnosed based on anamnesis and basic clinical signs. A relatively weak effect in men with early-onset male-pattern hair loss leads us to hypothesize that vitamin D should be given to these individuals in combination with other agents reducing thyroid antibody titers and improving thyroid functioning, especially with selenomethionine [25] and we intend to verify this question in our future study. Contrary to SPINA-GT, vitamin D had a neutral effect on Jostel’s thyrotropin index and SPINA-GD in both treatment arms [18, 19]. Therefore, exogenous vitamin D does not seem to affect the secretory function of human thyrotropes and does not modulate the activity of peripheral deiodinases, converting thyroxine into triiodothyronine.

The study protocol allows us only to hypothesize potential mechanisms explaining the obtained changes. Dimorphism in vitamin D action on antibody titers may be a consequence of differences in the androgen status of patients, as well as differences in the impact of cholecalciferol on free testosterone. Some of our observations support this explanation. The strength of vitamin D action on thyroid antibody titers depended on baseline levels of calculated free testosterone. Moreover, we have found correlations between baseline levels of total testosterone or DHEA-S and circulating titers of TPOAb. The lack of similar correlations for TgAb may be associated with the fact that they are less sensitive and less specific compared with TPOAb [27]. Furthermore, there was a clear difference between both groups in the impact of vitamin D on calculated free testosterone. Exogenous cholecalciferol resulted in a decrease in testosterone levels in subjects with normal hair and had a neutral effect in individuals with male-pattern hair loss. Interestingly, only testosterone-treated, but not testosterone-naïve, hypogonadal men have been characterized by an increase in testosterone levels after cholecalciferol treatment [15]. Therefore, it is possible that the association between the impact of vitamin D on testosterone production and baseline testosterone levels has an inverted U-shaped pattern.

Between-group differences in the strength of action of exogenous cholecalciferol on thyroid autoimmunity may also be partially attributed to disturbances in cholecalciferol homeostasis in individuals with early-onset androgenic alopecia. This hypothesis is based on the association between thyroid autoimmunity and low vitamin D status, as well as on a beneficial effect of cholecalciferol supplementation on thyroid antibody titers [25, 28]. In line with this hypothesis, both groups of our study differed in the impact of treatment on 25-hydroxyvitamin D levels, which was more pronounced in subjects with normal hair growth than in patients with alopecia. Moreover, the impact of exogenous vitamin D on TPOAb and TgAb titers correlated with treatment-induced changes in 25-hydroxyvitamin D. Theoretically, vitamin D supplementation may not have as much effect in individuals with early-onset alopecia, because, in spite of their lower 25-hydroxyvitamin D levels, they may have appropriate calcitriol levels which is more biologically active than vitamin D.

Yet another mechanism explaining our findings are differences in insulin sensitivity. Both study groups differed in baseline values of HOMA1-IR. Moreover, unlike in men with alopecia, exogenous vitamin D exerted a significant effect on this marker in subjects with normal hair growth and this effect correlated with the degree of reduction in titers of TPOAb and TgAb. Interestingly, other authors observed that in non-obese euthyroid individuals TPOAb titers were associated with HOMA1-IR and C-reactive protein levels, independent of thyroid function and these observations suggested that mild deviation of thyroid function within the normal range, chronic inflammation, and insulin resistance may be a link between thyroid autoimmunity and metabolic abnormalities in the non-obese population [29]. If this hypothesis is correct, the impact of exogenous cholecalciferol on thyroid autoimmunity may be potentiated by concomitant use of metformin known to affect hypothalamic-pituitary-thyroid axis activity [30].

Taking into account the measured parameters, the low size of the correlations between the outcome measures was an expected finding of the current study. The obtained results indicate that thyroid autoimmunity is, apart from vitamin D status, insulin sensitivity and sex hormones, regulated by many other factors (such as genetic factors, iodine and selenium intake, age, infections, radiation exposure and endocrine disruptors). These factors exert an external influence on thyroid antibody titers, thyroid function and androgen status and may modulate the impact of exogenous vitamin D on these parameters.

Other study limitations should also be addressed. The main drawback of the study is a small sample size, limiting the statistical significance of the obtained results. Although the study protocol minimized the impact of random diurnal, seasonal and analytical variations in the measured variables, we cannot rule out the regression-toward-the-mean effect. Because the area where the study was conducted was characterized by sufficient iodine supply [31] and low selenium status [32], it is uncertain whether the effect of vitamin D is the same in men living in iodine-deficient areas and/or in areas with adequate selenium intake. Finally, because of the inclusion criteria, the question whether there are differences in the action of vitamin D on antibody titers and thyroid function tests in hypothyroid patients with early-onset androgenic alopecia and normal hair growth remains unanswered.

Summing up, individuals with early-onset androgenic alopecia were characterized by higher titers of thyroid antibodies, correlating with androgen levels and 25-hydroxyvitamin D levels. Exogenous vitamin D reduced thyroid antibody titers in patients with early-onset hair loss to a lesser extent than in subjects with normal hair growth. This effect depended on the magnitude of an increase in 25-hydroxyvitamin D levels and the improvement in insulin sensitivity. The current study shows for the first time that euthyroid men with early-onset androgenic alopecia may benefit to a lesser degree from vitamin D treatment than other subjects with autoimmune thyroiditis. Because of numerous study limitations, our study should be regarded as a pilot one and should be verified in a large-scale prospective study.

## Abbreviations

DHEA-S: Dehydroepiandrosterone-sulfate
FSH: Follicle-stimulating hormone
HOMA1-IR: The homeostasis model assessment 1 of insulin resistance index
hsCRP: High sensitivity C-reactive protein
IU: International unit
LH: Luteinizing hormone
PCOS: Polycystic ovary syndrome
SD: Standard deviation
SPINA: Structure parameter inference approach
TgAb: Thyroglobulin antibodies
TPOAb: Thyroid peroxidase antibodies
